# Thoracoscopic enucleation of an esophageal glomus tumor in the prone position: a case report and literature review

**DOI:** 10.1186/s40792-024-01934-6

**Published:** 2024-05-28

**Authors:** Shigeki Matsumoto, Tomoyuki Okumura, Takeshi Miwa, Yoshihisa Numata, Takeru Hamashima, Miki Ito, Yasuhiro Nagaoka, Chitaru Takeshita, Ayano Sakai, Nana Kimura, Mina Fukasawa, Kosuke Mori, Naoya Takeda, Kenta Yagi, Ryo Muranushi, Takahiro Manabe, Yoshihiro Shirai, Toru Watanabe, Katsuhisa Hirano, Isaya Hashimoto, Kazuto Shibuya, Isaku Yoshioka, Tsutomu Fujii

**Affiliations:** 1 Department of Surgery and Science, Faculty of Medicine, Academic Assembly, University of Toyama, 2630 Sugitani, Toyama, 930-0194 Japan; 2 Office of Human Research Ethics, Faculty of Education and Research Promotion, Academic Assembly, University of Toyama, 2630 Sugitani, Toyama, 930-0194 Japan; 3 Department of Pathology, Faculty of Medicine, Academic Assembly, University of Toyama, 2630 Sugitani, Toyama, 930-0194 Japan

**Keywords:** Glomus tumor, Esophagus, Minimally invasive surgery, Thoracoscopic enucleation, Sengstaken–Blakemore (SB) tube

## Abstract

**Background:**

Glomus tumors (GT) generally occur in the skin. However, esophageal GT, an extremely rare condition, has no established standardized treatment guidelines. Herein, we report the case of an esophageal GT successfully removed by thoracoscopic enucleation in the prone position using intra-esophageal balloon compression.

**Case presentation:**

A 45-year-old man underwent an annual endoscopic examination and was found to have a submucosal tumor in the lower esophagus. Endoscopic ultrasound (EUS) revealed a hyperechoic mass originating from the muscular layer. Contrast-enhanced computed tomography identified a 2 cm mass lesion with high contrast enhancement in the right side of the lower esophagus. Pathologic findings of EUS-guided fine needle aspiration biopsy (EUS–FNA) revealed round to spindle shaped atypical cells without mitotic activity. Immunohistochemically, the tumor was positive for alpha-smooth muscle actin, but negative for CD34, desmin, keratin 18, S-100 protein, melan A, c-kit, and STAT6. He was diagnosed with an esophageal GT and a thoracoscopic approach to tumor resection was planned. Under general anesthesia, a Sengstaken–Blakemore (SB) tube was inserted into the esophagus. The patient was placed in the prone position and a right thoracoscopic approach was achieved. The esophagus around the tumor was mobilized and the SB tube balloon inflated to compress the tumor toward the thoracic cavity. The muscle layer was divided and the tumor was successfully enucleated without mucosal penetration. Oral intake was initiated on postoperative day (POD) 3 and the patient discharged on POD 9. No surgical complications or tumor metastasis were observed during the 1-year postoperative follow-up.

**Conclusions:**

As malignancy criteria for esophageal GT are not yet established, the least invasive procedure for complete resection should be selected on a case-by-case basis. Thoracoscopic enucleation in the prone position using intra-esophageal balloon compression is useful to treat esophageal GT on the right side of the esophagus.

## Background

Glomus tumors (GT) are mesenchymal tumors composed of modified smooth muscle cells that represent a neoplastic counterpart to the perivascular glomus bodies [1]. The majority of GT are found in subcutaneous tissues of the subungual region but also rarely in the gastrointestinal (GI) tract [1, 2]. In addition, the vast majority of GT found in the GI tract occur in the stomach, making esophageal GT extremely rare [1, 3]. Although almost all GT are benign, several malignant cases are reported [4] with a criterion for malignant GT in subcutaneous tissues proposed [5]. However, neither a grading criterion nor a standard treatment for GT in the GI tract is established. We report here the case of successfully esophageal GT removal by thoracoscopic enucleation in the prone position using intra-esophageal balloon compression.

## Case presentation

**Fig. 1:**
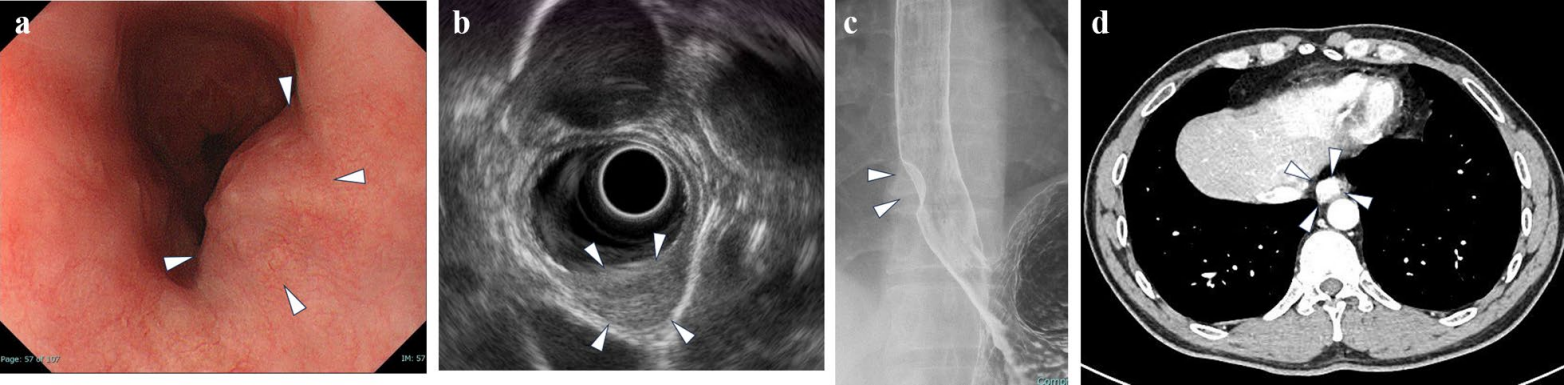
Preoperative findings. **a** Upper gastrointestinal endoscopy observed a submucosal lesion in the lower thoracic esophagus (white arrowhead). **b** EUS revealed a homogeneous hyperechoic mass originating from the submucosal layer (white arrowhead). **c** Upper GI series showed a submucosal mass in the lower thoracic esophagus (white arrowhead). **d** Contrast-enhanced CT image showed a mass lesion with high contrast enhancement in the right side of the lower esophagus (white arrowhead)

A 45-year-old man underwent an annual physical examination and was found to have a tumor in the lower esophagus without any symptoms. He had no significant medical or family history and unremarkable laboratory data. His upper gastrointestinal endoscopy revealed a 2 × 2 cm mass lesion with submucosal elevation in the lower thoracic esophagus (Fig. 1a). Endoscopic ultrasound (EUS) indicated a 1.6 × 2 cm homogeneous hyperechoic mass with a sharply demarcated smooth surface originating from the muscular layer (Fig. 1b). Upper GI series showed a submucosal mass in the lower thoracic esophagus (Fig. 1c). Contrast-enhanced computed tomography revealed a 2 × 2 cm mass lesion with homogeneous high contrast enhancement in the right side of the lower esophagus (Fig. 1d). There was no evidence of local invasion, lymph node involvement or distant metastasis. Fluorine-18 fluorodeoxyglucose–positron emission tomography (FDG–PET) showed no positive uptake in the tumor (data not shown).

**Fig. 2:**
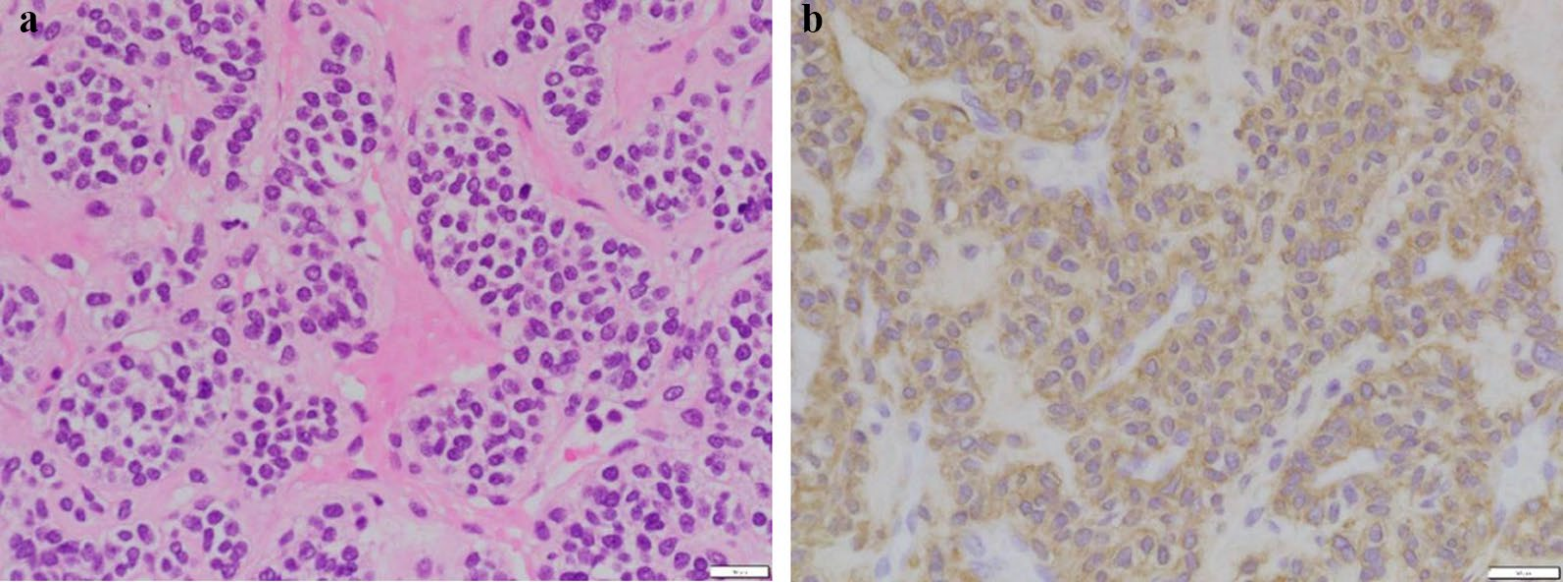
Pathologic diagnosis of EUS–FNA specimens. **a** HE staining (×200). **b** Immunohistochemical staining for alpha-smooth muscle actin (×200)

Pathologic diagnosis of EUS-guided fine needle aspiration biopsy (EUS–FNA) revealed linear, cordate, vesicular, and perivascular proliferation of atypical cells with foci of hyalinized and fibrous stroma. Atypical cells were small round to spindled shape with increased chromatin, chromatin granularity, and irregular nuclei (Fig. 2a). Mitotic activity was not detected. Immunohistochemical staining revealed that the tumor was positive for alpha-smooth muscle actin (Fig. 2b), but negative for CD34, desmin, keratin 18, S-100 protein, melan A, c-kit, and STAT6. Therefore, he was diagnosed with esophageal GT. Based on the tumor size, histological characteristics, and patient’s physical status, tumor resection by a minimally invasive thoracoscopic approach was planned.

**Fig. 3:**
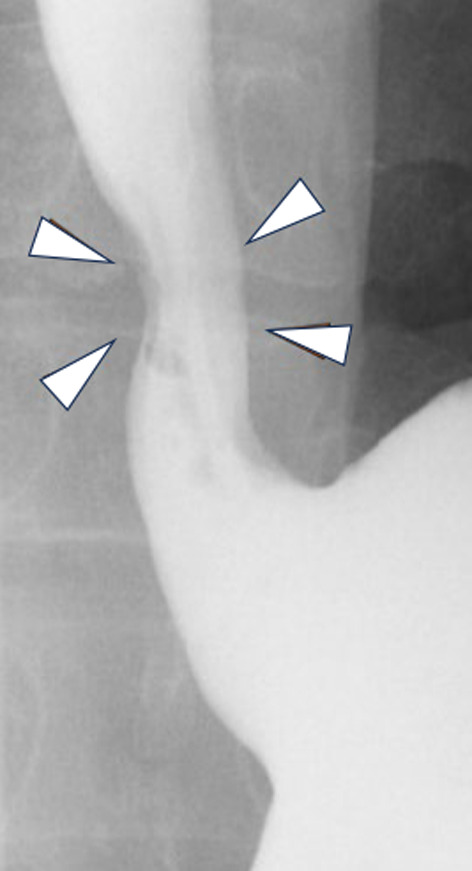
Postoperative fluoroscopy on POD 3 showed a smooth passage at the suture site (white arrowhead)

A Sengstaken–Blakemore (SB) tube was placed in the esophagus under general anesthesia. A right thoracoscopic approach was performed in the prone position under artificial pneumothorax. The tumor was identified on the right side of the lower esophagus. The esophageal balloon of the SB tube was inflated and the tumor was compressed towards the right thoracic cavity. The muscle layer was split and the elastic soft tumor was effectively extruded by the balloon pressure dilatation. The tumor was then successfully enucleated without mucosal penetration. The esophageal muscular layer was closed with a continuous barbed absorbable suture. The operation time was 184 min, and the total blood loss was uncountable. Postoperative fluoroscopy on postoperative day (POD) 3 showed a smooth passage at the suture site (Fig. 3), with oral intake started on the same day. He was discharged on POD 9 in good physical condition.

**Fig. 4:**
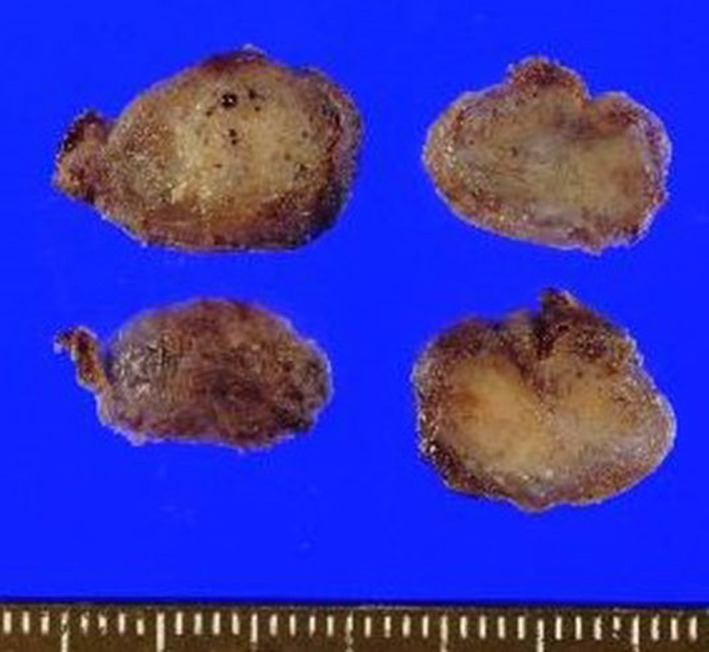
Macroscopic examination of the resected specimen showed that the tumor was 15 mm in size and remained within the capsule, with no apparent tumor exposure

Macroscopic examination of the resected and fixed specimen showed a tumor 15 mm in size, retained within the capsule with no apparent tumor exposure (Fig. 4).

Pathologic examination of the resected specimen revealed round to spindle shaped tumor cells with atypical round nuclei and eosinophilic to pallid cytoplasm. Mitosis or vascular invasion was not detected. Immunohistochemical staining revealed that the tumor cells were diffusely positive for alpha-smooth muscle actin. In addition to molecules such as CD34, desmin, keratin 18, S-100 protein, melan A, c-kit, and STAT6 that were examined in the preoperative biopsy specimen, AE1/AE3, synaptophysin, and DOG1 were all negative in the resected specimen. These findings were consistent with preoperative histologic examination (Fig. 2a, b), further confirming the diagnosis of GT.

No surgical complications such as leakage, stenosis or reflux, and no tumor metastasis were observed during the 1-year postoperative follow-up.

## Discussion

GT is composed of cells resembling modified smooth muscle cells of the normal glomus body, which is involved in temperature regulation via arteriovenous shunting of blood [6]. Though most GTs are usually found in the skin of the hand, they are also reported in the deep soft tissue, bone, lung, and GI tract [1, 2, 4, 5, 7]. However, the vast majority of GI tract GT occur in the stomach, with esophageal GT an extremely rare occurrence [1, 3].

To date, 12 cases of esophageal GT are reported in English, including the present case [2, 3, 8–16]. They are summarized in Table 1. Among the cases described for each of the tumor characteristics, 5 are male and 7 are female, respectively. The average age of the 12 cases was 50.3 years old (range 28–79). Of the 11 cases, tumors were located as follows: 4 in the lower esophagus, 4 in the middle esophagus, 2 in the upper esophagus, and 1 in the cervical esophagus. The mean tumor size of the 11 cases was 4.4 cm (range 1.5–8.0). Three out of 12 cases had lymph node and/or distant metastasis.

**Table 1:** Review of available English case reports of esophageal glomus tumors

Case No	Author	Year of publication	Ref. No	Age	Sex	Location	Size (cm)	Nuclear atypia	Mitotic activity	Cell shape	Vascular involvement	Metastasis	Treatment	Surgical procedure
1	Papla B	2001	[8]	79	F	Upper	5	None	None	NA	NA	None	Surgery	Exploratory thoracotomy
2	Altorjay A	2003	[9]	41	F	Upper	5	None	None	Round	NA	None	Surgery	Enucleation via cervical approach
3	Tomas D	2006	[10]	28	F	Lower	3	None	2/50HPF	Round	None	None	Surgery	Partial esophagectomy with gastroesophageal anastomosis
4	Zhang Y	2013	[11]	47	M	Middle	5.8	Present	10/50HPF	Round	Present	Lymph node	Surgery	Ivor–Lewis esophagectomy
5	Bali GS	2013	[12]	49	F	Lower	7.6	NA	9/50HPF	Spindle /Round	None	None	Surgery	Minimally invasive esophagectomy with partial gastrectomy
6	Nishida K	2013	[2]	69	M	Middle	2.1	Present	2/50HPF	Spindle /Round	NA	None	Surgery	Enucleation through right thoracotomy
7	Segura S	2015	[13]	66	F	Cervical	3	None	None	Round	NA	None	None	
8	Ugras N	2015	[14]	47	F	Lower	8	Present	6/50HPF	Spindle /Round	NA	None	Surgery	Ivor–Lewis esophagectomy through right thoracotomy
9	Marcella C	2019	[3]	30	M	Middle	2	None	12% Ki67 index	Round	NA	None	Endoscopic resection	
10	Seban RD	2020	[15]	45	M	NA	NA	NA	NA	NA	NA	Liver, lung, mediastinum, bone, skin	Surgery/CT/RT	NA
11	Xiao A	2022	[16]	57	F	Middle	5	Present	15/50HPF	Spindle /Round	NA	Lung, lymph node	RT	
12	Matsumoto S	2024	Our case	45	M	Lower	1.5	Present	None	Spindle /Round	None	None	Surgery	Thoracoscopic enucleation

NA: not available; CT: chemotherapy; RT: radiation therapy; HPF: high power fields

Although most GTs are benign, some malignant cases are reported [5]. Folpe et al. proposed the following criteria for metastatic GT of the peripheral soft tissue: deep location, size greater than 2 cm, atypical mitotic figures, moderate to high-grade nuclear atypia, and five or more mitoses per 50 HPF [5]. While Miettinen et al. proposed a size greater than 5 cm, spindle cell changes, and vascular involvement, but not mitotic activity or nuclear atypia, as criteria for malignant gastric GTs [1]. However, for esophageal GT, criteria for malignancy are not reported due to the extreme rarity of cases and limited follow-up.

Based on the summary of 12 cases of esophageal GTs (Table 1), the presence of multiple features such as larger size, severe nuclear atypia, higher mitotic activity, and vascular invasion appear linked to an increased metastasis risk. However, it is still difficult to clearly distinguish esophageal malignant GTs by histological means. Spindle cells are found in many esophageal GTs regardless of metastases. Therefore, robust malignancy criteria for esophageal GT are not yet established, with the tumor in the present case determined of uncertain malignant potential. Considering that the histological characteristics (small tumor size, absence of severe atypia, mitotic figures, or vascular involvement) were not strongly suggestive of metastatic potential, and that the patient was 45 years old with good surgical tolerance, minimally invasive tumor enucleation was performed rather than subtotal esophagectomy with lymph node dissection or long-term imaging surveillance.

Complete surgical excision is recommended for GT of the peripheral soft tissue [17], and partial gastrectomy, also known as wedge gastrectomy, with adequate free margins but without lymphadenectomy is suggested as appropriate treatment for GT of the stomach [2, 7, 18]. Endoscopic or laparo-endoscopic combined resection are also reported for small gastric GT [18]. Among the esophageal GTs listed in Table 1, endoscopic resection was performed in one case with a tumor 2 cm in size. Enucleation was performed in 3 cases with tumor sizes of 1.5 cm, 2.1 cm, and 5 cm. Partial esophagectomy was performed in one case with a tumor 3 cm in size. Esophagectomy was performed in 3 cases with tumor sizes of 5.8 cm, 7.6 cm, and 8 cm. These summaries suggested that the least invasive procedure for complete resection was selected on a case-by-case basis depending on tumor size and location.

Recently, thoracoscopic enucleation in the prone position was widely adopted for benign submucosal tumors (SMT) of the esophagus, providing optimal visualization of the surgical field and better perioperative outcomes [19]. Specific for SMT in the right esophageal wall, the intra-esophageal balloon compression method is reported to allow safe tumor enucleation, resulting in expulsion of the tumor from the esophageal wall [20]. In the case of tumor occurring on the left side of the esophageal wall, hybrid surgery combined with endoscopic and thoracoscopic approaches may be considered [21]. Robot-assisted thoracoscopic approach is also considered useful in some cases [22].

## Conclusions

A 1.5 cm esophageal GT found on the right side of the lower esophagus without strongly suggestive malignant features, was successfully removed by thoracoscopic enucleation in the prone position incorporating intra-esophageal balloon compression.

As esophageal GT malignancy criteria are not yet established, the least invasive procedure for complete resection should be selected on a case-by-case basis depending on the size, location, and histologic tumor characteristics. Careful follow-up after surgery is also recommended.

## Data Availability

The data sets of this case report are available from the corresponding author upon reasonable request.

## Abbreviations

GT: Glomus tumor
GI: Gastrointestinal
EUS: Endoscopic ultrasound
CT: Computed tomography
FDG–PET: Fluorine-18 fluorodeoxyglucose positron emission tomography
FNA: Fine needle aspiration biopsy
SB: Sengstaken–Blakemore
ICS: Intercostal space
POD: Postoperative day
